# Predicting COVID-19 Incidence Using Anosmia and Other COVID-19 Symptomatology: Preliminary Analysis Using Google and Twitter

**DOI:** 10.1177/0194599820932128

**Published:** 2020-06-02

**Authors:** Bharat A. Panuganti, Aria Jafari, Bridget MacDonald, Adam S. DeConde

**Affiliations:** 1 Department of Surgery, Division of Otolaryngology–Head and Neck Surgery, University of California–San Diego, La Jolla, California, USA; 2 Department of Otolaryngology–Head and Neck Surgery, Harvard Medical School, Boston, Massachusetts, USA; 3 Department of Otolaryngology–Head and Neck Surgery, Massachusetts Eye and Ear Infirmary, Boston, Massachusetts, USA; 4 School of Medicine, University of California–San Diego, La Jolla, California, USA

**Keywords:** COVID-19, Google trends, Twitter, infodemiology, epidemiology

## Abstract

**Objective:**

To determine the relative correlations of Twitter and Google Search user trends concerning smell loss with daily coronavirus disease 2019 (COVID-19) incidence in the United States, compared to other severe acute respiratory syndrome coronavirus 2 (SARS-CoV-2) symptoms. To describe the effect of mass media communications on Twitter and Google Search user trends.

**Study Design:**

Retrospective observational study.

**Setting:**

United States.

**Subjects and Methods:**

Google Search and “tweet” frequency concerning COVID-19, smell, and nonsmell symptoms of COVID-19 generated between January 1 and April 8, 2020, were collected using Google Trends and Crimson Hexagon, respectively. Spearman coefficients linking each of these user trends to COVID-19 incidence were compared. Correlations obtained after excluding a short timeframe (March 22 to March 24) corresponding to the publication of a widely read lay media publication reporting anosmia as a symptom of infection was performed for comparative analysis.

**Results:**

Google searches and tweets concerning all nonsmell symptoms (0.744 and 0.761, respectively) and COVID-19 (0.899 and 0.848) are more strongly correlated with disease incidence than smell loss (0.564 and 0.539). Twitter users tweeting about smell loss during the study period were more likely to be female (52%) than users tweeting about COVID-19 more generally (47%). Tweet and Google Search frequency pertaining to smell loss increased significantly (>2.5 standard deviations) following a widely read media publication linking smell loss and SARS-CoV-2 infection.

**Conclusions:**

Google Search and tweet frequency regarding fever and shortness of breath are more robust indicators of COVID-19 incidence than anosmia. Mass media communications represent important confounders that should be considered in future analyses.

There has been considerable attention in the news media and medical literature regarding smell loss as a potential early manifestation of severe acute respiratory syndrome coronavirus 2 (SARS-CoV-2) infection. On March 22, 2020, for example, the *New York Times* published a widely read article describing the mounting evidence of this association.^1^ Moreover, in a recently published study of 237 patients, wherein clinicians were surveyed regarding patient symptomatology using the coronavirus disease 2019 (COVID-19) Anosmia Reporting Tool (developed by the American Academy of Otolaryngology–Head and Neck Surgery), 73% of patients reported anosmia prior to COVID-19 diagnosis, and 26.6% reported smell loss as the heralding symptom.^2^

With the current need for real-time epidemiological data, social media and Internet user behavior may be uniquely suited to advise COVID-19-related resource allocation and mitigation strategies. In a recent study by Walker et al,^3^ for example, Google search frequency (Google Trends) pertaining to smell loss was correlated both with COVID-19 disease and mortality. This study used the power of user-generated content in an electronic medium to study public health trends, also known as infodemiology. Indeed, the relative distinctiveness of anosmia as a symptom, particularly compared to other COVID-19 symptoms (ie, cough, fever, shortness of breath, and/or fatigue), may offer unique, temporally sensitive data related to SARS-CoV-2 infection and may be worthy of infodemiological investigation.

However, conclusions regarding disease trends based on social media or Internet search data are inferred, not definitive. A recent and well-known application of infodemiology, Google Flu Trends, for example, sought to predict regional spikes in influenza but was shuttered after consistent overprojections.^4^ The method of deriving the search terms and the media’s influence on user behavior were cited reasons for failure and represent an important reason to exercise caution when using this type of data.^5^ As such, although significant correlations between Google searches pertaining to anosmia and COVID-19 incidence have already been reported, our intention in the present study is to better understand the relative value of alternative infodemiological parameters (nonsmell symptoms, COVID-19 searches and tweets) and platforms (Twitter) in estimating COVID-19 infection trajectory in the United States. Twitter, as a social media platform, allows for real-time research into user-generated opinions, feelings, and health status with concomitant demographic data. Twitter, therefore, may serve as an important adjunct to Google Search user trends in infodemiological investigations. Its use during the COVID-19 pandemic, however, has not yet been reported.

In this study, we detail our findings following a preliminary infodemiological exploration into COVID-19 incidence and its correlation with multiple user trends in an online forum. Specifically, we sought to (1) investigate Twitter “tweets” as an alternative or adjunct to Google Trends to understand COVID-19 incidence patterns, (2) elucidate the relative infodemiological value of Google searches and tweets regarding smell loss compared to nonsmell COVID-19 symptoms, and (3) understand the influence of news media on infodemiological trends related to smell loss.

## Methods

### Twitter

Twitter data, including tweet frequency and inferred Twitter user demographic information (sex and age), were cultivated using Crimson Hexagon, a web-based social media library and analysis platform that allows review of all publicly available tweets filterable by search terms, location, and date range. We used search terms concerning COVID-19 (“COVID,”“coronavirus,”“COVID-19,”“SARS-CoV2,” and “COVID19”), nonsmell symptoms of COVID-19 (“shortness of breath,”“fatigue,”“cough,” and “fever”), and smell loss (“anosmia,”“loss of smell,”“reduced smell,”“decreased smell,”“lose your sense of smell,”“lost sense of smell,”“decreased sense of smell,”“decrease your sense of smell,”“decreased my sense of smell,”“reduce your sense of smell,”“reduced my sense of smell,”“reduced sense of smell,”“loss of sense of smell,”“loss of smell,”“hyposmia”) to collect Twitter-derived data concerning smell loss and COVID-19. We excluded “retweets,” tweet replies, and tweets containing URLs in the primary analysis to better understand user behavior and to avoid capturing more passive mass media communications. In Crimson Hexagon, Twitter user information (age and sex) is derived from an algorithm accounting for variables including author interests, time since registration, and Twitter users the user follows. The reported accuracy of inferred age assignment is 65% and is classified in 1 of 4 age groups (<18, 18-24, 24-34, and ≥35).^6^

### Google Trends

Information pertaining Google Search trends was obtained using Google Trends, an open-access platform that provides normalized search frequency data (scale of 0-100). We extracted search frequency data concerning loss of smell (search terms included “loss of smell,”“anosmia,”“lose smell,”“sense of smell,”“cannot smell,”“can’t smell,” and “hyposmia”), COVID-19 (same as Twitter), nonsmell symptoms of COVID-19 (same as Twitter), and a commonly prescribed therapy for smell loss (“nasal irrigation” and “sinus rinse”). Frequency of searches about dysgeusia (“dysgeusia,”“taste change,” and “taste loss”) was also obtained and correlated to COVID-19 incidence. Search terms enclosed in quotations automatically included queries with words before and after the first and last words, respectively, of the enclosed phrase.

### Data Analysis

The specified time period for both Crimson Hexagon and Google Trends queries was January 1, 2020, through April 8, 2020, to encompass a “control period” for tweets and searches before COVID-19 cases were diagnosed in the United States. Only data from the United States were included in the present analysis to mitigate potential confounding effects (eg, variability of Twitter use or Google searches internationally) and inappropriate exclusions or inclusions of search terms borne from incorrect translation or nonaccounting of regional vernacular in this word-based study. Data pertaining to daily COVID-19 case numbers in the United States were collected from the *New York Times* administered repository of COVID-19–related case data.^7^ Data concerning COVID-19 incidence and Google Search and tweet frequencies were examined individually via histograms and then in conjunction via scatterplots, revealing nonnormal distributions and nonlinear correlations. As such, Spearman rank correlation coefficients were obtained to assess the relationship between Google Search and tweet frequency, as well as daily incidence of COVID-19 in the United States. Fisher *r*-to-*z* transformations were performed to compare Spearman correlations. A *P* value of less than .05 was considered the threshold for significance. Last, to understand tweet and Google Search trends in relation to mass media communications, we characterized “peaks” in tweet and Google Search frequency as being at least 2 standard deviations above their mean over the study period.

Correlations between COVID-19 incidence and Google Search frequency pertaining to smell loss and nonsmell COVID-19 symptoms from a similar time period (January 1, 2019, through April 8, 2019) in 2019 were obtained to confirm that infodemiological trends in COVID-19 symptoms were indeed unique to the COVID-19 era.

## Results

### Tweets and COVID-19 Incidence

**Figure 1.**
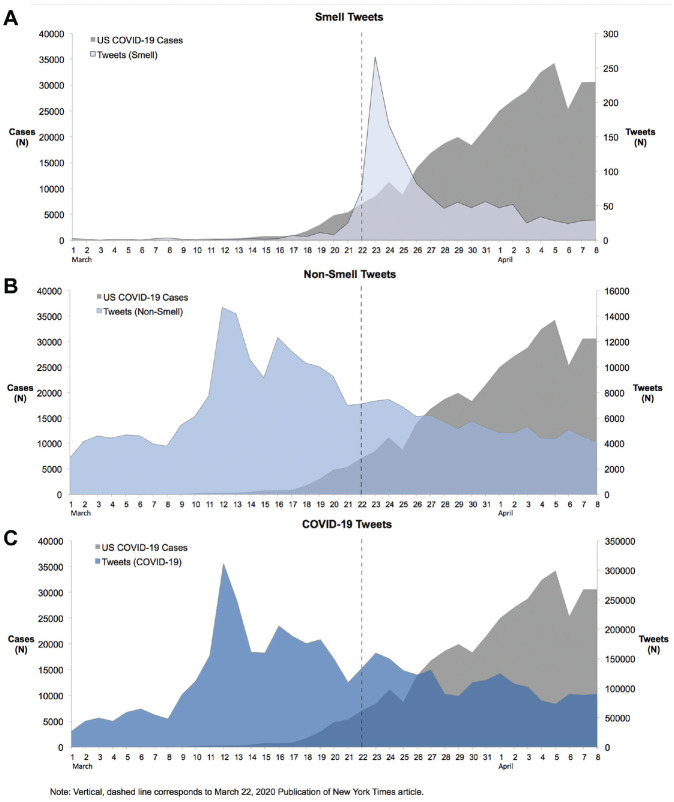
Frequency curves of coronavirus disease 2019 (COVID-19) incidence in the United States and tweets concerning (A) loss of smell, (B) nonsmell symptoms, and (C) COVID-19. The vertical line represents the date of lay article linking anosmia with COVID-19. Vertical, dashed line corresponds to March 22, 2020, publication of *New York Times* article.

Tweet frequency concerning smell loss (0.539) was not as well correlated with daily COVID-19 incidence as tweet frequency concerning COVID-19 (0.848), nonsmell symptoms (0.761), and both and smell and nonsmell symptoms together (0.765) (**Tables 1** and **2**). A significant peak in tweets concerning smell loss (>3 standard deviations greater than the mean for smell tweets) was seen around a widely read *New York Times* article reporting a link between anosmia and COVID-19 infection (March 22, 2020)^1^ (see Suppl. Table SA in the online version of the article); data pertaining to March 22, 2020, and the 2 following days were excluded in 1 iteration of the analysis to help evaluate quantitatively the effect of discrete, lay media transmissions on Twitter and Google search trend correlations with COVID-19 incidence. While the Spearman correlation pertaining smell tweets (0.498) decreased, it remained statistically significant; more incremental decreases were seen in the correlation coefficients concerning nonsmell symptom (0.756). The change in the anosmia tweet correlation was not significant, however (*P* = .349). Moreover, using Twitter, we had the unique ability to identify tweets containing URLs and retweets. The total number of tweets with URLs and retweets included was 42,924, compared to 1444 when they were excluded, representing a 97% difference in tweet frequency (see Suppl. Table SA in the online version of the article). In addition, when excluding March 22 to 24, 2020, the correlation coefficient linking smell loss tweets (when including URLs, retweets, and replies) improved significantly from 0.240 to 0.553 (*P* = .004) (**Table 1** and **Figure 1**).

**Table 1.** Spearman Correlation Coefficients Relating Tweets and Google Searches With COVID-19 Incidence Between January 1 and April 8, 2020, in the United States.^a^

Parameter	Entire Study Duration	*P* Value	Excluding March 22-24	*P* Value	Difference	*P* Value
Tweets about anosmia	0.539	<.001	0.483	<.001	−0.056	.299
Tweets about anosmia with URLs, retweets, and replies included	0.240	.016	0.553	<.001	0.313	.004
Tweets about nonsmell symptoms	0.761	<.001	0.756	<.001	−0.005	.467
Tweets about all symptoms (anosmia and nonsmell symptoms)	0.765	<.001	0.760	<.001	−0.005	.467
Tweets about COVID-19	0.848	<.001	0.851	<.001	0.003	.470
Google searches about anosmia	0.564	<.001	0.524	<.001	−0.040	.346
Google searches about cough	0.629	<.001	0.612	<.001	−0.017	.424
Google searches about fatigue	0.052	.611	0.065	.531	0.013	.464
Google searches about shortness of breath	0.732	<.001	0.716	<.001	−0.016	.407
Google searches about fever	0.749	<.001	0.739	<.001	−0.010	.438
Google searches about all nonsmell symptoms	0.744	<.001	0.733	<.001	−0.011	.433
Google searches about nasal rinses or sinus irrigations	0.307	.002	0.272	.007	−0.035	.395
Google searches about COVID-19	0.899	<.001	0.893	<.001	−0.006	.416
Google searches about dysgeusia	0.512	<.001	0.467	<.001	−0.045	.341
Google searches about anosmia from 2019	−0.223	.027				

Abbreviation: COVID-19, coronavirus disease 2019.

a Significance of differences between Spearman correlations were assessed after performing Fisher *r*-to-*z* transformation.

Of Twitter users who posted about smell loss, 52% were reported to be female, compared to 47% of all users posting about COVID-19 (*P* < .001). However, reported age distributions among users tweeting about smell loss (80% were 35 or older) were similar to the reported age distributions among users tweeting about COVID-19 (78% were 35 or older).

### Google Searches and COVID-19 Incidence

**Figure 2.**
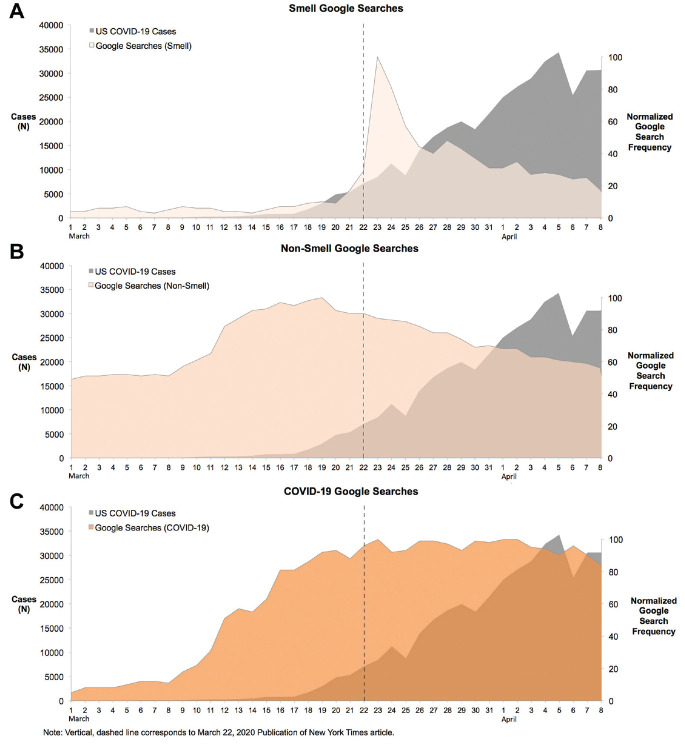
Frequency curves of coronavirus disease 2019 (COVID-19) incidence and Google Search trends concerning (A) loss of smell, (B) nonsmell symptoms, and (C) COVID-19. The vertical line represents the date of lay article linking anosmia with COVID-19. Vertical, dashed line corresponds to March 22, 2020, publication of *New York Times* article.

Spearman correlations between Google Search queries concerning smell loss, COVID-19, nonsmell symptoms, common anosmia therapies (“nasal irrigations,”“sinus rinse”), and daily COVID-19 incidence were similarly assessed. The correlation between COVID-19 incidence and searches pertaining smell loss (0.524) was less robust than searches about nonsmell symptoms (0.744) and COVID-19 (0.893). There was no difference between dysgeusia (0.512) and smell loss correlations with COVID-19 incidence. Spearman correlations between daily COVID-19 incidence and each individual nonsmell symptom were also assessed. Both fever (0.749) and shortness of breath (0.732) were better correlated with COVID-19 incidence than anosmia (**Table 2**). No correlation was seen between fatigue search frequency and disease incidence (0.052; *P* = .611). When excluding the time period from March 22 to March 24, 2020, we again saw, although not statistically significant, a change in the anosmia search correlation (0.523 from 0.564). The new correlation with smell loss (0.523) remained significant. Search frequency regarding nasal irrigations or sinus rinses (0.307; *P* = .002) was only weakly correlated with COVID-19 incidence (**Table 1** and **Figure 2**).

**Table 2.** Comparison of Twitter and Google Search Spearman Correlation Coefficients.

Parameter	Difference	*P* Value^a^
Google Search vs Twitter		
Smell loss	0.025	.401
Nonsmell symptoms	−0.017	.392
COVID-19	0.051	.064
Google Search parameter vs smell loss		
Cough	−0.065	.241
Fever	−0.185	.010
Shortness of breath	−0.168	.020
Nonsmell symptoms	0.180	.013
Nasal rinse/sinus irrigation	−0.257	.011
COVID-19	0.335	<.001
Twitter parameter vs smell loss		
Nonsmell symptoms	0.222	.003
All symptoms (including smell)	0.226	.002
COVID-19	0.309	<.001

Abbreviation: COVID-19, coronavirus disease 2019.

a Significance of differences between Spearman correlations were assessed after performing Fisher *r*-to-*z* transformation.

### Twitter vs Google Search Parameter Correlations With COVID-19 Incidence

No significant differences between tweet and Google Search correlations corresponding to smell loss (*P* = .401), all nonsmell symptoms (*P* = .392), COVID-19 (*P* = .064), and COVID-19 incidence were observed (**Table 2**).

## Discussion

The COVID-19 pandemic has affected millions of people in over 183 countries or territories, with wide-ranging sociopolitical and economic consequences.^8^ While investigations into treatment methods are ongoing, there have been concomitant efforts to understand disease manifestations and patterns of spread using Internet and social media platforms. The recognition of smell loss as a potential heralding and discriminant symptom of SARS-CoV-2 infection is impactful in both spreading awareness and as a potential temporally sensitive infodemiological tool to inform disease trajectory research.^9^

In a timely publication by Walker et al,^3^ a significant correlation was identified between anosmia search volume (0.636) and COVID-19 incidence in the United States between January 1 and March 25, 2020. The correlation reported in our study (0.564), while significant, was less strong, which may reflect a slight difference in study period. Importantly, we found that Google searches and tweets concerning COVID-19, shortness of breath, fever, and all nonsmell symptoms combined were more strongly correlated with COVID-19 incidence, which suggests that nonsmell loss–related parameters may be more sensitive to COVID-19 incidence than anosmia (**Table 2**). This could be related to the fact that despite it being a distinctive symptom, the reported frequency of smell loss associated with COVID-19 across the extant literature is variable but may be much lower than the other symptoms.^10,11^ Moreover, as smell loss has been reported to be associated with a milder disease course, patients with a smell loss phenotype may be less likely to be tested for COVID-19 and thus underrepresented in overall incidence.^12^ Therefore, it is plausible that anosmia may indeed be a sensitive infodemiological parameter for COVID-19 incidence in the setting of more widespread testing. We also investigated dysgeusia separately, as it may represent more significant smell loss. However, there was no significant difference between dysgeusia (0.512) and smell loss correlations with COVID-19 incidence.

We also postulated that Twitter might offer additional or unique insight into user health status as Twitter represents a more expressive medium, allowing users to their post about their opinions, concerns, and symptoms.^13^ However, correlations derived from tweets pertaining to smell dysfunction, nonsmell symptoms, and COVID-19 were similarly sensitive to COVID-19 incidence to their corresponding Google Searches (**Table 2**). More granular analysis may have allowed us to narrow our search to tweets explicitly relating to user reports of smell loss and improved the sensitivity of our correlations, but the natural language processing required was beyond the scope of the present study. We did, however, have the unique ability to gather user-specific demographic information with Twitter. Interestingly, a greater proportion of users tweeting about smell loss were female (52%), although a lesser proportion of users tweeting about COVID-19 were female (47%). While this may simply reflect sex-based differences in care-seeking behavior, which has been demonstrated across a broad range of conditions, this trend might also offer putative insight regarding sex-oriented discrepancies in COVID-19 presentation.^14^

A significant spike in anosmia-related searches and tweets was seen after March 22, 2020, corresponding precisely to the publication of a widely read *New York Times* article linking anosmia and SARS-CoV-2 infection (see Suppl. Table SA in the online version of the article). To quantify the effect of mass media communications on the correlation between smell loss tweets, searches, and COVID-19 incidence, we excluded March 22 to March 24 to obtain new Spearman correlation coefficients. Only small, nonsignificant reductions in correlation were observed after excluding these 3 days. Moreover, we aimed to mitigate the influence of mass media communications by excluding tweets containing URLs and retweets, which we hypothesized were more likely to reflect user responses to media than their personal experiences. In analyzing tweets about smell loss that contained URLs and retweets, however, we identified a significant improvement in the Spearman coefficient linking anosmia tweets and COVID-19 incidence (0.240 to 0.553) (**Table 1**). This illustrates the significant potential influence in infodemiological data that can be introduced by mass media communication. As such, researchers must demonstrate an awareness of such influence when interpreting user-generated data in the context of understanding COVID-19 disease trends.

Herein, we present a preliminary analysis illustrating the potential use of both Twitter and Google Trends user data as potential corollaries for COVID-19 incidence. While we identified several interesting relationships, we also highlight some pitfalls of infodemiological investigations in this rapidly evolving media-sensitive setting. First, we found that Google Search and tweet frequency concerning COVID-19, and not anosmia or any other COVID-19 symptom described in other infodemiological investigations, had the strongest correlation with daily disease incidence in the United States. Moreover, we clearly found that mass media communication played a significant role in driving user behavior in both mediums (Twitter and Google Search). This is a potential confounder of user-generated data that must be carefully accounted for in similar infodemiological inquiries. In addition, the background seasonal variation in other viral illnesses with similar symptomatology queried here, including influenza, could have affected the results. We suspect, however, that this influence is small given the overwhelming incidence of COVID-19 relative to other viral illnesses. We hope that these preliminary findings and lessons learned may be levied to inform and enhance future COVID-19–related studies using infodemiological methods. To that end, we recognize the possibility of future changes in findings borne from a more developed understanding of SARS-CoV2 prevalence (accounting for the rate of asymptomatic carriership, for example).

## Conclusions

Google Search and tweet frequency regarding more common COVID-19 symptomatology (ie, fever and shortness of breath) are more robust indicators of daily disease incidence than anosmia. Although Twitter represents an alternative platform for infodemiological investigations, tweet frequency pertaining to COVID-19–related symptoms was similar in sensitivity to Google Search trends. Last, mass media communications represent important confounders that must be considered when correlating infodemiological trends with COVID-19 incidence.

## Supplemental Material

SUPPLEMENTARY_TABLE – Supplemental material for Predicting COVID-19 Incidence Using Anosmia and Other COVID-19 Symptomatology: Preliminary Analysis Using Google and Twitter Click here for additional data file. Supplemental material, SUPPLEMENTARY_TABLE for Predicting COVID-19 Incidence Using Anosmia and Other COVID-19 Symptomatology: Preliminary Analysis Using Google and Twitter by Bharat A. Panuganti, Aria Jafari, Bridget MacDonald and Adam S. DeConde in Otolaryngology–Head and Neck Surgery
