# Supplementary figures and images for: Predicting COVID-19 Incidence Using Anosmia and Other COVID-19 Symptomatology: Preliminary Analysis Using Google and Twitter
Source: Otolaryngol Head Neck Surg. 2020 Jun 2;163(3):491–7. doi: 10.1177/0194599820932128 (PMC7267744; doi:10.1177/0194599820932128)

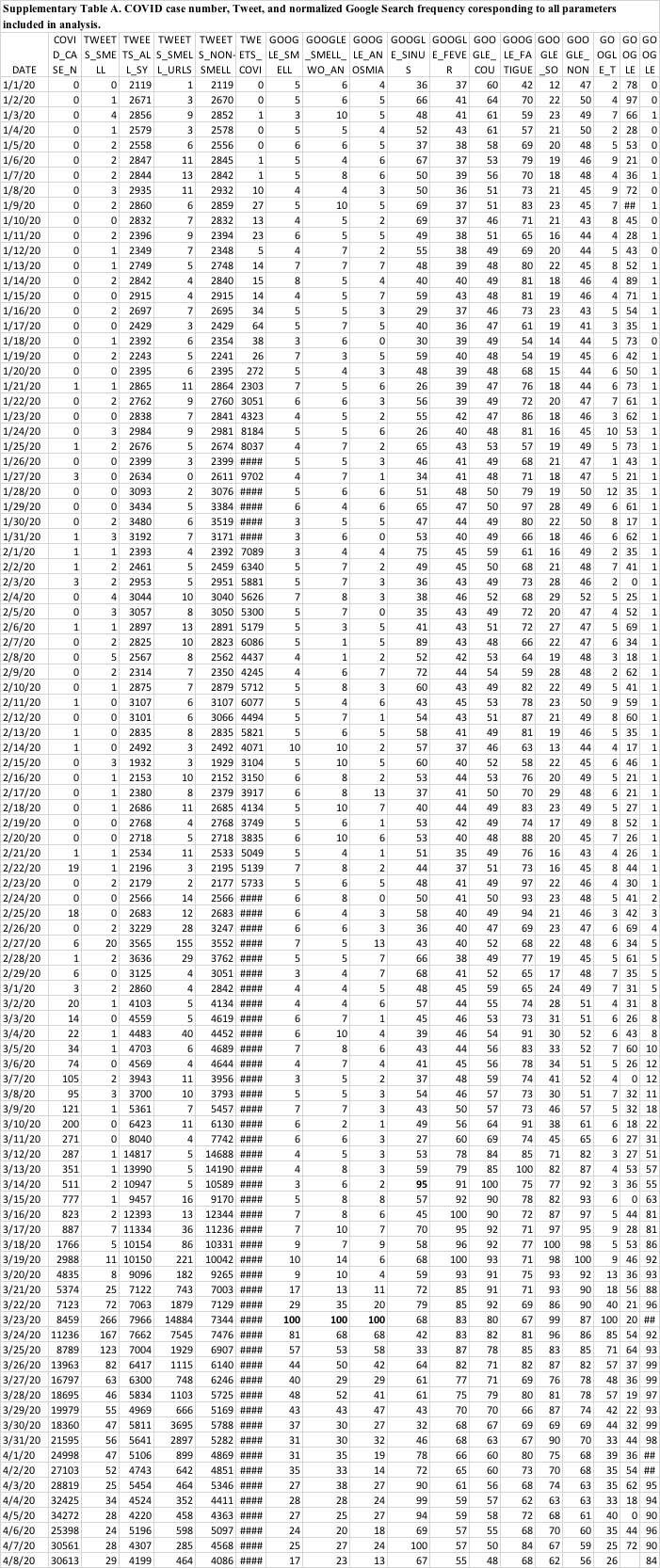

Supplement: SUPPLEMENTARY_TABLE – Supplemental material for Predicting COVID-19 Incidence Using Anosmia and Other COVID-19 Symptomatology: Preliminary Analysis Using Google and Twitter [file SUPPLEMENTARY_TABLE.docx]
